# Differences in the Phenolic Composition and Antioxidant Properties between *Vitis coignetiae* and *Vitis vinifera* Seeds Extracts

**DOI:** 10.3390/molecules18033410

**Published:** 2013-03-14

**Authors:** Stanisław Weidner, Anna Rybarczyk, Magdalena Karamać, Angelika Król, Agnieszka Mostek, Joanna Grębosz, Ryszard Amarowicz

**Affiliations:** 1 Department of Biochemistry, University of Warmia and Mazury, Oczapowskiego Street 1A, Olsztyn 10-957, Poland; E-Mails: weidner@uwm.edu.pl (S.W.); anna.rybarczyk@umb.no (A.R.); angelikakrol@op.pl (A.K.); aga.mostek@wp.pl (A.M.); grebosz.joanna@gmail.com (J.G.); 2 Institute of Animal Reproduction and Food Research of the Polish Academy of Sciences, Tuwima Street 10, 10-747 Olsztyn, Poland; E-Mail: m.karamac@pan.olsztyn.pl

**Keywords:** grapevine seeds, catechins, phenolic acids, antioxidant activity

## Abstract

Phenolic compounds were extracted from European and Japanese grapevine species (*Vitis vinifera* and *V. coignetiae*) seeds using 80% methanol or 80% acetone. The total content of phenolic compounds was determined utilizing Folin-Ciocalteu’s phenol reagent, while the content of tannins was assayed by the vanillin and BSA precipitation methods. Additionally, the DPPH free radical and ABTS cation radical scavenging activities and the reduction power of the extracts were measured. The HPLC method was applied to determine the phenolic compounds, such as phenolic acids and catechins. The seeds contained large amounts of tannins and gallic acid and observable quantities of catechins, *p*-coumaric, ferulic and caffeic acids. The dominant form of phenolic acids in the extracts was the ester-bound form. The content of total phenolics was higher in the European grape *V. vinifera* seeds, which also contained more tannins, catechins and phenolic acids, except for caffeic acid. Extracts from *V. vinifera* seeds showed better radical scavenger properties and stronger reducing power. The total contents of phenolic compounds and tannins in acetone extracts were higher than in methanolic extracts. Acetone extracts also exhibited stronger antiradical properties as well as stronger reducing power.

## 1. Introduction

In recent years, researchers have been paying more attention to plant polyphenols. Grape seeds, which are a by-product of wine and fruit juice production, contain about 5–8% polyphenolics, mainly flavonoids [1]. They are a rich source of proanthocyanidins; *i.e.*, condensed tannins, which are composed of polymeric units of flavan-3-ol monomers: (+)-catechin, (−)-epicatechin and (−)-epicatechin gallate [2]. These compounds are present in seeds, especially in seed coats, and their considerable quantities are extracted during the production of red wine, affecting its sensory properties [2,3].

Gallic acid, catechins (*i.e.*, catechin, epicatechin, epigallocatechin, epicatechin gallate, epigalocatechin gallate), procyanidin B_1_, and procyanidin B_2_ were the dominant low-molecular-weight polyphenolic constituents in *Vitis vinifera* seed extract [4]. The presence of monomers, dimers, trimers, tetramers, and pentamers of catechin in *Vitis vinifera* seeds was reported by Simonetti *et al*. [5]. Polymers P_6_, P_7_, P_8_, and P_9_ were determined in the extract from *Vitis vinifera* seeds by Peng *et al*. [6].

Polyphenols are bioactive components in the human diet. The anticarcinogenic effect of polyphenols is attributed to their antioxidant properties, as well as their capability to modulate the activity of enzymes, block hormone receptors and lower the activity of mutagens. Polyphenols also protect blood vessels, reduce the aggregation of blood platelets and lower the LDL-cholesterol level in blood [7]. Once the positive health effects of proanthocyanidins were recognized, grape seeds extracts began to be marketed as food supplements/nutraceuticals [8,9]. Proanthocyanidins can also be added to food products as natural antioxidants to extend their shelf life and replace currently added synthetic compounds, such as BHA and BHT, suspected to have a carcinogenic effect [10,11].

Determination of the chemical composition of secondary metabolites frequently lies at the foundation of higher plant taxonomy, because every species and every taxonomic unit is characterized by a certain, species-specific composition of these compounds [12]. The diversity of phenolic substances produced by plants as well as the toxicity of many phenol oxidation or hydrolysis products make researchers claim that phenolic compounds are the plant’s response to stresses, both biotic and abiotic ones [13,14,15,16].

The main objective of the experiment was to compare the content of phenolic compounds in seeds of the Japanese grapevine *Vitis coignetiae*, and the European *Vitis vinifera.* We have also compared the antioxidant properties of the extracts obtained from the seeds.

## 2. Results and Discussion

### 2.1. Content of Total Phenolics

The content of total phenolics compounds in grape seeds was determined by the colorimetric method using Folin-Ciocalteu’s phenol reagent. The results of these determinations are presented in Table 1. The grapevine species *Vitis vinifera* was characterized by a much higher content of phenolics in the seeds based on fresh weight. The results for *V. vinifera* are as follows: 21.21 (methanolic extraction) and 30.02 mg/g FW (acetone extraction). Compared to the results obtained for *V. coignetiae*, these values are 8.99 mg/g FW (methanolic extraction) and 12.81 mg/g FW (acetone extraction) higher. These analyses also demonstrated that acetone extracts from seeds of both analyzed varieties of grapevine containe a higher total quantity of phenolic compounds than methanolic preparations. The differences in the total content of phenolics between methanolic and acetone extracts obtained from seeds of the same vine species reached 55.99 mg/g of extract for *V. coignetiae* and 98.2 mg/g extract for *V. vinifera*. This difference was, therefore, more prominent in the case of extracts from the European grapevine.

**Table 1:** Content of total phenolics in the seeds and their Total Antioxidant Capacity.

Solvent used for extraction	Species	Total phenolics (mg/g FW)	TEAC (mmol Trolox/g FW)
80% methanol	*Vitis coignetiae*	8.99 ± 0.06 ^a^	0.071 ± 0.002 ^a^
	*Vitis vinifera*	21.21 ± 0.05 ^b^	0.152 ±0.002 ^b^
80% acetone	*Vitis coignetiae*	12.81 ± 0.16 ^a^	0.094 ± 0.002 ^a^
	*Vitis vinifera*	30.02 ± 0.99 ^b^	0.196 ± 0.002 ^b^

For the same solvent means with the same letter are not significantly different (*p* < 0.05).

The analyses performed in this research have enabled us to detect large quantities of phenolic compounds in the seeds of the species *V. coignetiae* and *V. vinifera*. It has been demonstrated that seeds of the European grapevine contained significantly more of these compounds. The results of the analysis on the total content of phenolics in seeds of *V. vinifera*, obtained in the present study, are similar to those reported by Baydar *et al*. [17] for the same grape species. Our earlier experiments proved that the total content of phenolics in seeds of *V. vinifera* is much higher than in seeds of *V. amurensis* and similar to that in seeds of *V. californica* and *V. riparia* [18]. It should be added that the total content of phenolics in the seeds of *V. coignetiae* assayed in the present study is similar to that found in the seeds of *V. amurensis* [18].

### 2.2. Content of Condensed Tannins

In order to determine the content of condensed tannins in the acetone and methanolic extracts, two methods were used: the vanillin assay and the of precipitation of tannins with BSA protein. The results of these determinations can be seen in Table 2. It was demonstrated that all of the extracts were capable of forming bonds with the BSA protein and of precipitation [18]. The content of tannins in the extracts of *V. coignetiae* seeds determined by the vanillin assay was 0.305 and 0.371 A_500_/mg of methanolic and acetone extract, respectively. For comparison, the content of tannins in extracts from *V. vinifera* seeds was 0.697 and 0.817 A_500_/mg of methanolic and acetone extracts, respectively. The results produced with the other method; *i.e.*, the BSA precipitation of tannins, were as follows: 0.223 and 0.288 A_510_/mg of, respectively, methanolic and acetone extracts from *V. coignetiae* seeds and 0.467 and 0.566 A_500_/mg of, respectively, methanolic and acetone extracts from *V. vinifera* seeds. Both tests have shown that the extracts of *V. vinifera* are rich in tannins. For extracts of both *V. coignetiae* and *V. vinifera* seeds, a higher content of tannins were detected in the acetone extracts.

**Table 2:** Content of condensed tannins in the grapevine seed extracts.

Solvent used for extraction	Species	Protein precipitation method (Absorbance at 510 nm/mg)	Vanillin/HCl method (Absorbance at 500 nm/mg)
80% methanol	*Vitis coignetiae*	0.223 ± 0.002 ^a^	0.305 ± 0.004 ^a^
	*Vitis vinifera*	0.467 ± 0.005 ^b^	0.697 ± 0.005 ^b^
80% acetone	*Vitis coignetiae*	0.288 ± 0.007 ^a^	0.371 ± 0.003 ^a^
	*Vitis vinifera*	0.566 ± 0.005 ^b^	0.817 ± 0.005 ^b^

For the same solvent means with the same letter are not significantly different (*p* < 0.05)

The results of this research coincide with those reported by many authors, who have shown that grape seeds are a rich source of proanthocyanidins, which can also occur as ester bonds with gallic acid [3,7,9,10,19]. Comparing the present results with those obtained by Weidner *et al*. [18], it has been revealed that the content of tannins in the seeds of *V. coignetiae* is similar to that in the seeds of *V. californica* and *V.* *riparia*, whereas the seeds of *V. vinifera* contained much more of tannins than the former three grapevine species.

### 2.3. UV Spectra

**Figure 1:**
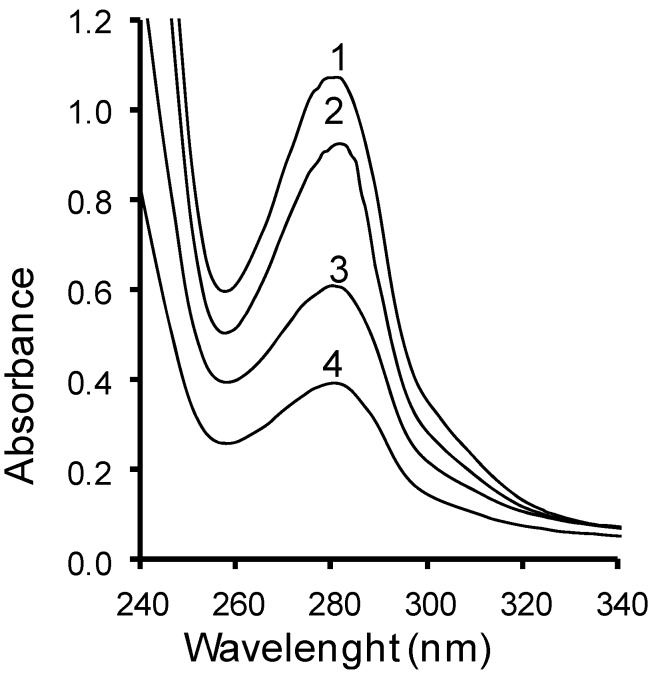
UV spectra of grapevine seed extracts.

The UV spectra of the phenolic compounds derived from acetone and methanolic extracts of grapevine seeds are depicted in Figure 1. In the UV spectra of phenolic compounds from methanolic and acetone extracts from the seeds of *V. coignetiae*, absorption maxima were observed at a wavelength of 280 nm, most probably originating from tannins, catechins, and gallic acid. Due to a low content of phenolic acids in the extracts, no maxima were observed in the longer wavelengths (295–320 nm) region.

(1) acetone extract of *V.vinifera*; (2) methanolic extract of *V.vinifera*; (3) acetone extract of *V. coignetiae*; (4) methanolic extract of *V. coignetiae.*

### 2.4. Antioxidant Activity

Table 1 contains the results of our assays with the free-radical cation of 2,2'-azinobis-(3-ethylbenzothiazoline-6-sulfonic acid) (ABTS^+•^), which establishes the Trolox equivalent antioxidant capacity (TEAC) of methanolic and acetone extracts from seeds of *V. coignetiae* and *V. vinifera*. The solution of ABTS^+•^ for this analysis is blue and green in colour and attains a maximum absorbance at a wavelength of 734 nm. When a solution of antioxidants is introduced, ABTS^+•^ disappears, which is evidenced as a loss of colour of the reagent mixture. The analysis was performed in order to detect and quantify the antioxidant power of the analyzed extracts. It was determined that the extracts from seeds of *V. vinifera* had a statistically higher percent inhibition of the absorbance of the ABTS^+•^ solution than those from seeds of *V. coignetiae*. The TEAC antioxidant capacity expressed in mM Trolox eq./g of extract for methanolic and acetone extracts from seeds of *V. vinifera* was greater by 1.24 and 1.37 mM Trolox eq./g, respectively, than that for extracts of *V. coignetiae*. It was noted demonstrated that acetone extracts from seeds of both grapevine species have a greater capacity of scavenging ABTS^+•^ free-radical cations, thus possessing a higher antioxidant capacity (Trolox equivalent antioxidant capacity or TEAC).

**Figure 2:**
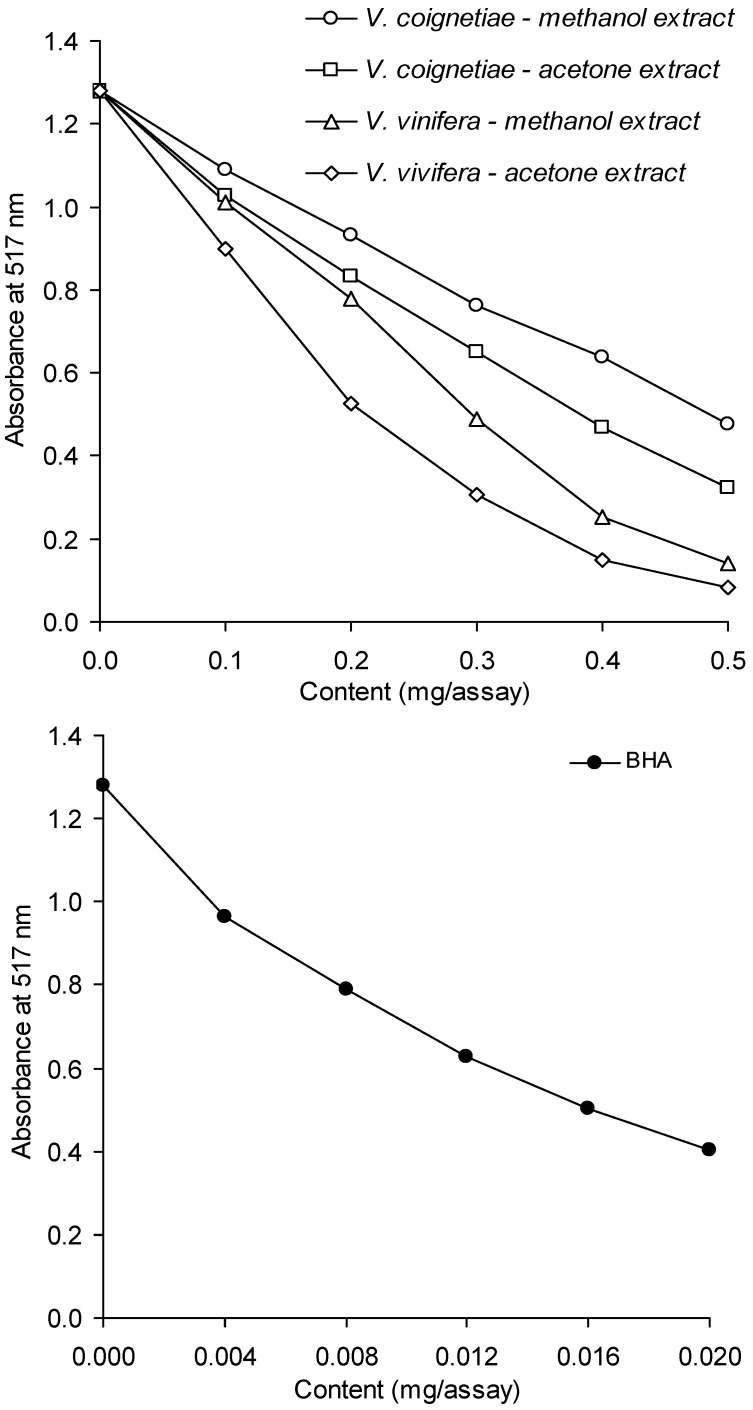
Antiradical activity of extracts of grapevine seeds and BHA against DPPH free-radical.

The results of tests on the capacity of methanolic and acetone extracts of *V. coignetiae* and *V. vinifera* seeds for scavenging DPPH free-radicals are listed in Figure 2. A solution of DPPH (2,2-diphenyl-1-picrylhydrazyl) free-radical, deep purple in colour and with a maximum absorbance at a wavelength of 517 nm, was used for this analysis. The colour of DPPH^•^ solution disappears in the presence of antioxidants. It is therefore possible to monitor the reduction of DPPH free-radicals by observing a decline in absorbance readings. Our determinations were carried out in order to find out whether, and to what extent, the analyzed extracts demonstrated antioxidant properties. The results indicate that extracts of *V. vinifera* seeds produce a much stronger antioxidant efficacy. At a concentration of 0.1–0.5 mg of extract per sample, had a 2.5 to 3.3-fold higher capacity for the scavenging of DPPH^•^ was observed compared to extracts from seeds of *V. coignetiae*. Another observation was that acetone extracts were more powerful DPPH^•^ scavengers than methanolic counterparts. When the concentration of an analyzed sample was within 0.02 and 0.05 mg of the extract, the methanolic and acetone extracts from seeds of both grapevine species were statistically significantly different in their capacity for scavenging DPPH^•^. Noteworthy is the fact that within the above range of concentrations, the results obtained for particular samples differed in a statistically significant fashion. The investigated extracts were much weaker DPPH^•^ scavengers than butylated hydroxyanisole (BHA).

**Figure 3:**
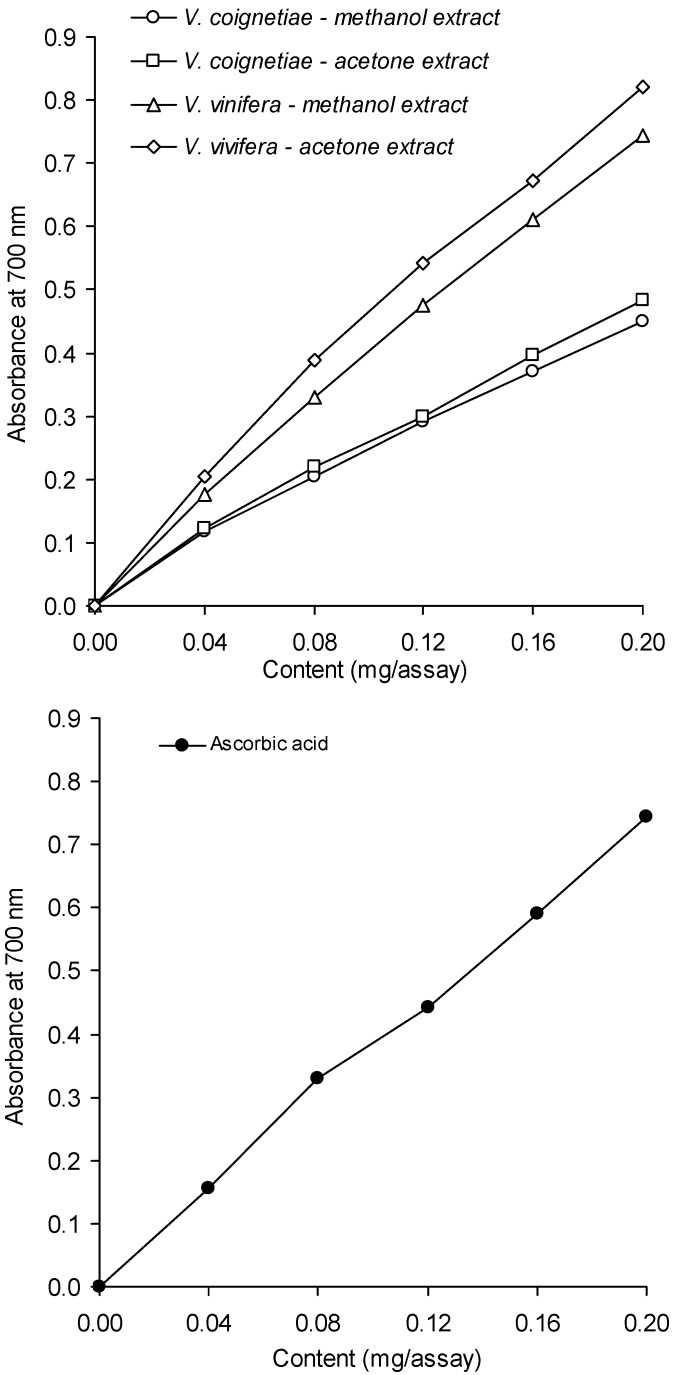
Reducing power of the extracts of grapevine seeds and ascorbic acid.

The results of our analysis for the reducing power of methanolic and acetone extracts from seeds of *V. coignetiae* and *V. vinifera* are presented in Figure 3. By this analytical method, the antioxidants present in the analyzed extracts reduced Fe^3+^ originating from potassium ferrocyanide to the divalent form, Fe^2+^; a processes which causes the appearance of a colour compound known as Prussian Blue. Prussian Blue yields a maximum absorbance at a wavelength of 700 nm. Extracts from the seeds of *V. vinifera* exhibited a much stronger antioxidant effect than those from seeds of *V. coignetiae*. This effect was similar to that of ascorbic acid at the same concentration.

The reducing power of extracts from seeds of *V. vinifera*, at a concentration of the analyzed sample equal to 0.20 mg of extract/sample, was about 1.7-fold higher than that of extracts from seeds of *V. coignetiae*. Statistically significant differences in the reducing power between extracts from the seeds of the European grapevine and extracts from seeds of *V. coignetiae* were demonstrated at concentrations for these samples within the range of 0.04 and 0.20 mg of extract/sample. 

Significantly greater reducing powers of acetone extracts compared to methanolic ones from the seeds of *V. vinifera* *V. coignetiae* was demonstrated at concentrations within 0.04–0.20 and 0.16–0.20 mg of extract/sample, respectively.

Extracts from the seeds of *V. vinifera* and *V. coignetiae* demonstrated a marked antioxidant power. All of the analyzed extracts were capable of scavenging the DPPH free-radical and ABTS cation radical. They also showed reducing power. Having analyzed all of the results of the assays conducted, it was concluded that the antioxidant properties of the extracts were strongly dependent on their content of phenolic compounds. The higher the content of phenolics, the greater antioxidant efficacy of the extracts. The positive correlation between the total content of phenolic compounds and antioxidant activity has been reported in earlier papers [20,21,22].

The strong antioxidant effect of the analyzed extracts was most probably the result of a high content of proanthocyanidins, monomeric flavan-3-ols as well as large quantities of gallic acid in these extracts, because the extracts from seeds of *V. vinifera*, which had more of the above compounds, were also stronger antioxidants. This conclusion is in accord with the numerous references on antioxidant properties of proanthocyanidins as well as (+)-catechin and (−)-epicatechin. As Shi *et al*. [1] state, the antioxidant power of these proanthocyanins is 20-fold stronger than that of vitamin E and 50-fold stronger than that of vitamin C. Da Silva *et al.* [23] demonstrated high activity of (+)-catechin and (−)-epicatechin as well as proanthocyanidins in scavenging DPPH free-radicals and the superoxide anion radical. They also demonstrated that flavan-3-ol monomers are much weaker free-radical scavengers that their polymers, and that gallic acid esterification invariably enhances this property in both cases. Another compound which reveals antioxidant properties is gallic acid. It owes its antioxidant power to the presence of as many as three hydroxyl groups in its structure, attached directly to the aromatic ring [1,7].

Jayaprakasha *et al*. [10], in their investigations on the antiradical activity of extracts from the seeds of *V. vinifera*, discovered a higher DPPH free-radical scavenging activity in acetone extract than in a methanolic one. Our results confirm the conclusion that acetone extracts of the analyzed grape seeds are stronger antioxidants than methanolic ones. This finding can be explained by the fact discovered and verified by other researchers that application of an aqueous solution of acetone during extraction yields more proanthocyanidins, thus resulting in better antioxidant properties of the extracts [10,24].

Extracts from seeds of *V. vinifera* proved to have a greater reducing power and higher DPPH free-radical scavenging activity compared to previously analyzed extracts from seeds of *V. riparia*, *V. amurensis* and *V. californica* [18]. Extracts from seeds of the other analyzed grape species, *V. coignetiae*, were comparable in the above properties to extracts from seeds of *V. californica* and *V. riparia* [18]. With this observation, it is possible to associate the antioxidant properties with the content of tannins in extracts, because extracts from the seeds of *V. vinifera* had the highest content of these compounds and therefore possessed the strongest antioxidant properties compared to those from *V. coignetiae* or extracts analyzed in the earlier experiment [18]. It needs to be added that extracts from the seeds of *V. coignetiae* possessed a comparable amount of tannins as extracts from seeds of *V. californica* and *V. riparia*, and they all demonstrated similar antioxidant properties.

The analyzed extracts from seeds of *V. vinifera* and *V. coignetiae* were also characterized by a much stronger reducing power and DPPH^•^ scavenging activity than, for example, extracts from roasted seeds of sesame, soybean, pumpkin or wheat germs [25]. Likewise, the Trolox equivalent antioxidant capacity (TEAC), and consequently the capability of scavenging ABTS radical monocation of the extracts analyzed by Kosińska and Karamać [25] was several-fold lower than that of the grape seed extracts analyzed in our experiment. It is worth mentioning that the reducing power and the DPPH free-radical scavenging activity demonstrated by the extracts from seeds of *V. vinifera* and *V. coignetiae* were higher than those in extracts from different types of tea [26].

### 2.5. Content of Catechins and Phenolic Acids

**Figure 4:**
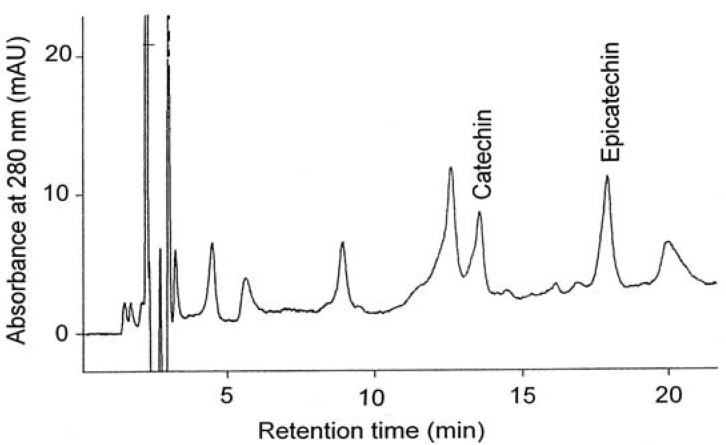
HPLC chromatogram of the grape seed catechins.

**Figure 5:**
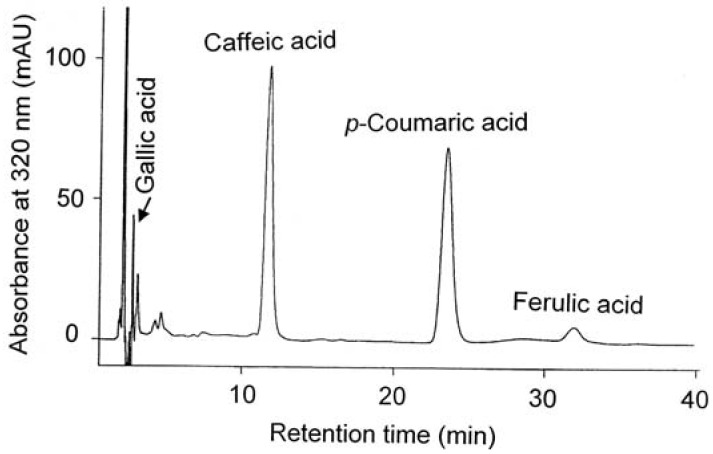
HPLC chromatogram of the grape seed phenolic acids liberated from the ester bounds.

The typical HPLC chromatograms of catechins and phenolic acids present in the grape seed extracts are depicted in Figure 4 and Figure 5.

The results of assays on the content of catechins in the seeds of *V. coignetiae* and *V. vinifera* are presented in Table 3. In seeds of the European grapevine, the content of catechin-group compounds was 0.98 mg/g FW when methanol was used for extraction and 1.07 mg/g FW for acetone extraction. For comparison, seeds of *V. coignetiae* contained 0.61 and 0.74 mg/g FW in methanolic and acetone extracts, respectively. These results suggest that seeds of *V. vinifera* possessed a higher amount of these compounds. They also indicate that catechins predominate over epicatechins in seeds of both analyzed grapevine species.

**Table 3:** Content of catechins in the grapevine seeds.

Solvent used for extraction	Species	(+)-Catechin (mg/g FW)	(−)-Epicatechin (mg/g FW)	(+)-Catechin + (−)-epicatechin (mg/g FW)
80% methanol	*Vitis coignetiae*	0.32 ± 0.02 ^a^	0.29 ± 0.01 ^a^	0.61 ± 0.18 ^a^
	*Vitis vinifera*	0.53 ± 0.03 ^b^	0.45 ± 0.02 ^b^	0.98 ± 0.05 ^b^
80% acetone	*Vitis coignetiae*	0.38 ± 0.02 ^a^	0.36 ± 0.03 ^a^	0.74 ± 0.04 ^a^
	*Vitis vinifera*	0.60 ± 0.03 ^b^	0.47 ± 0.03 ^b^	1.07 ± 0.06 ^b^

For the same solvent means with the same letter are not significantly different (*p* < 0.05).

The results of determinations of the content of gallic and *p-*coumaric acids in the seeds of *V. coignetiae* and *V. vinifera* are presented in Table 4. A much higher total content of gallic acid was demonstrated in the seeds of *V. vinifera* than those of *V. coignetiae*. The total content of gallic acid was 0.77 and 1.06 mg/g FW of seeds for the European grapevine and 0.25 and 0.44 mg/g FW of seeds for the Japanese species when using methanolic and acetone extractions, respectively. In all analyzed seeds, the dominant form of gallic acid was its ester-bound form. Differences in the total content of gallic acid were mainly attributable to the differences in the content of the ester-bound form. Seeds of *V. vinifera* contained 0.66 and 0.86 mg/g FW, while those of *V. coignetiae* had 0.18 and 0.35 mg/g FW of ester-bound gallic acid determined with methanol or acetone, respectively. Free and glycoside-bound gallic acid appeared in much smaller quantities (below 0.1 mg/g FW) in the analyzed seeds, and it was only the content of free gallic acid (in acetone extracts) that was slightly above this level, namely 0.13 mg/g FW. These results suggest that acetone extracts are characterized by a significantly higher total content of gallic acid than methanolic counterparts.

**Table 4:** Content of gallic and *p-*coumaric acids in the grapevine seeds.

Phenolic acid	Solvend used for extraction	Species	Form of phenolic acid	Content (mg/g FW)
Gallic	80% methanol	*Vitis coignetiae*	Free	35 ± 1 ^a^
			Esterified	180 ± 6 ^a^
			Glucosided	37 ± 1 ^a^
			Total	252 ± 8 ^a^
		*Vitis vinifera*	Free	51 ± 0.2 ^b^
			Esterified	665 ± 19 ^b^
			Glucosided	59 ± 2 ^b^
			Total	774 ± 23 ^b^
	80% acetone	*Vitis coignetiae*	Free	38 ± 1 ^a^
			Esterified	355 ± 12 ^a^
			Glucosided	47 ± 2 ^a^
			Total	439 ± 13 ^a^
		*Vitis vinifera*	Free	121 ± 3 ^b^
			Esterified	860 ± 28 ^b^
			Glucosided	76 ± 2 ^b^
			Total	1057 ± 35 ^b^
*p-*Coumaric	80% methanol	*Vitis coignetiae*	Free	1.56 ± 0.24 ^a^
			Esterified	3.97 ± 0.26 ^a^
			Glucosided	0.35 ± 0.05 ^a^
			Total	5.88 ± 0.55 ^a^
		*Vitis vinifera*	Free	3.25 ± 0.49 ^b^
			Esterified	10.58 ± 0.53 ^b^
			Glucosided	traces
			Total	13.83 ± 1.02 ^b^
	80% acetone	*Vitis coignetiae*	Free	-
			Esterified	5.67 ± 0.53 ^a^
			Glucosided	0.49 ± 0.09 ^a^
			Total	6.16 ± 0.26 ^a^
		*Vitis vinifera*	Free	-
			Esterified	11.24 ± 0.87 ^b^
			Glucosided	traces
			Total	11.24 ± 0.87 ^b^

For the same phenolic acid, solvent and form means with the same letter are not significantly different (*p* < 0.05).

The content of *p*-coumaric acid in the analyzed seeds, compared to that of gallic acid, was much lower; for example, 90-fold lower in seeds of *V. vinifera* extracted with acetone. Seeds of *V. vinifera* contained 13.83 and 11.24 µg/g FW of the total content of *p*-coumaric acid determined in the methanolic and acetone extracts, respectively. Seeds of *V. coignetiae* had 5.88 and 6.16 µg/g FW of the total content of *p*-coumaric acid in extracts obtained with methanol or acetone, respectively. The ester-bound form of this phenolic acid has been observed to prevail significantly in all of the analyzed extracts. In the extracts from seeds of *V. vinifera*, *p*-coumaric acid occurred exclusively as an ester-bound compound. Free *p*-coumaric acid was extracted only when methanol was applied. Noteworthy is the fact that seeds of *V. vinifera* were found to contain trace amounts of glycoside-bound *p*-coumaric acid, whereas in the extracts from seeds of *V. coignetiae*, these quantities were small but detectable: 0.34 (methanolic extracts) and 0.49 μg/g FW (acetone extracts). It has also been found that a higher total content of *p*-coumaric acid occurred in methanolic vs. acetone extracts from the seeds of *V. vinifera*. The content of this acid in both types of extracts from *V. coignetiae* seeds remained on a similar level and did not differ in a statistically significant manner.

Table 5 gives the results of our analysis of the content of caffeic and ferulic acids in methanolic and acetone extracts from seeds of *V. coignetiae* and *V. vinifera*. In all of the analyzed seeds, only the presence of the bound form of caffeic acid was determined. A higher content of this acid was detected in the seeds of *V. coignetiae*, which had 3.78 and 3.36 µg/g FW of caffeic acid in the methanolic and acetone extracts, respectively. In turn, the seeds of *V. vinifera* contained 2.73 and 2.35 µg/g FW in methanolic and acetone extracts, respectively. Ferulic phenolic acid appeared exclusively in the grape seed extracts as an ester-bound compound. Compared to the other analyzed phenolic acids, ferulic acid appeared in the smallest quantities. The lowest content of ferulic acid was detected in *V. vinifera* seeds: 1.95 and 2.35 µg/g FW in methanolic or acetone extracts, respectively. Comparing the content of ferulic acid in the seeds of *V. coignetiae*, its level was determined to be 0.86 µg/g FW in methanolic extract and only trace quantities of this compound were detected in the acetone extract.

**Table 5:** Content of the esterified caffeic and ferulic acids acids in the grapevine seeds.

Phenolic acid	Solvent used for extraction	Species	Content (μg/g FW)
Caffeic	80% methanol	*Vitis coignetiae*	3.78 ± 0.08 ^a^
		*Vitis vinifera*	2.73 ± 0.42 ^a^
	80% acetone	*Vitis coignetiae*	3.36 ± 0.43 ^a^
		*Vitis vinifera*	2.35 ± 0.25 ^a^
Ferulic	80% methanol	*Vitis coignetiae*	0.86 ± 0.14 ^a^
		*Vitis vinifera*	1.95 ± 0.17 ^a^
	80% acetone	*Vitis coignetiae*	trace
		*Vitis vinifera*	2.35 ± 0.31 ^a^

For the same solvent means with the same letter are not significantly different (*p* < 0.05).

Apropos the content of flavan-3-ol monomers: the levels (+)-catechin and (−)-epicatechin for the grape species investigated in this work proved to be much lower than those previously detected in seeds of *V. amurensis*, *V. californica* or *V. riparia* [18]. The concentrations of (+)-catechin and (−)-epicatechin in seeds of *V. vinifera* detected in our study were likewise lower than those reported by Kennedy *et al*. [19] for seeds of the same species (but obtained from fruit harvested in January). The difference, however, was rather small and most probably caused by a much longer extraction time applied in the cited research. The percentage of (+)-catechin and (−)-epicatechin in the extracts from seeds of *V. vinifera* was comparable to the finding of Saito *et al*. [8] for extracts from seeds of the same species. The determinations reported in this study revealed that quantities of (+)-catechin were slightly higher than those of (−)-epicatechin in the analyzed extracts of grape seeds. These results are in accordance with those reported by Escribano-Bailón *et al.* [27], who analyzed seeds of the same grape species.

For seeds of *V. vinifera* and *V. coignetiae* analyzed in the present investigation, considerable quantities of gallic acid as well as smaller amounts of *p*-coumaric, caffeic and ferulic acids were determined. The presence of gallic acid in the extracts from seeds of *V. vinifera* was also demonstrated by other authors [19,27]. Many researchers state that this acid occurs in ester bonds with flavan-3-ol monomers as (+)-catechin 3-*O*-gallate and in bonds with flavan-3-ol polymers [1,2,27]. This observation agrees with the results of the present study, as most of the gallic acid determined in the grape seed extracts appeared in the ester-bound form. Both gallic and *p*-coumaric acids had also been detected previously in the extracts from seeds of *V. amurensis*, *V. californica* and *V. riparia* [18]. Analogously to the present experiment, these acids occurred mainly in the ester-bound form and gallic acid largely prevailed over *p*-coumaric acid. It is noteworthy that seeds of *V. vinifera* contained more of both gallic and *p*-coumaric acids than the seeds of *V. amurensis*, *V. californica* or *V. riparia*. The percentage of gallic acid in the extracts from seeds of *V. vinifera* analyzed in the present study was similar to that reported by Shafiee *et al*. [19] for extracts from seeds of the same grape species.

## 3. Experimental

### 3.1. Chemicals

All solvents used were of analytical grade unless otherwise specified. Methanol, acetone, hexane, acetonitrile, potassium ferricyanide, and trichloroacetic acetic (TCA) were acquired from the P.O.Ch. Company (Gliwice, Poland). Vanillin, Folin & Ciocalteu’s phenol reagent, bovine serum albumin (BSA), triethanolamine (TEA), ascorbic acid, butylated hydroxyanisole (BHA), sodium dodecyl sulfate (SDS), 2,2'-azinobis-(3-ethylbenzothiazoline-6-sulfonic acid) (ABTS), 6-hydroxy-2,5,7,8-tetramethyl-chroman-2-carboxylic acid (Trolox), 2,2'-diphenyl-1-picrylhydrazyl radical (DPPH**^•^**), (+)-catechin, (−)-epicatechin, gallic acid, caffeic, acid, *p*-coumaric acid, and ferulic acid were obtained from Sigma-Aldrich (Poznań, Poland).

### 3.2. Plant Material

*Vitis coignetiae* and *Vitis vinifera* (var. Pinot noir) seeds were supplied by Sandeman Seeds (Lalongue, France).

### 3.3. Extract Preparation

Seeds were ground in a coffee mill and defatted with hexanes in a Soxhlet apparatus at 50 °C for 6 h. Phenolic compounds were then extracted from raw material under nitrogen using 80% (w/v) acetone or methanol at a solids to solvent ratio of 1:10 (w/v), at 80 °C for 15 min [28]. The extraction was repeated twice more, the supernatants were filtrated and combined, and the organic solvent was evaporated under vacuum at 45 °C; the remaining aqueous solution was lyophilized.

### 3.4. Content of Total Phenolics

The content of total phenolics in the crude extract was estimated using Folin & Ciocalteu’s phenol reagent [29]. (+)-Catechin was used as a standard in this work.

### 3.5. Determination of Condensed Tannins by the Vanillin Method

The content of condensed tannins in the extracts was determined using a modified vanillin assay [30]. The results were expressed as absorbance units at 500 nm per mg of the extract (A_500_/mg). Briefly, to 1.0 mL of the extract, 5.0 mL of solution B (obtained by dissolving 0.5 g vanillin in 100 mL of solution A) was added. Solution A was made by adjusting 4 mL of concentrated HCl to 100 mL with methanol. The samples were left in the dark at room temperature for 20 min, and then absorbance was measured at 500 nm.

### 3.6. Determination of Condensed Tannins by the Protein Precipitation Method

The content of tannins in the extracts was determined according to Hagerman and Butler [31]. Briefly, 2 mL of a bovine serum albumin (BSA) solution (concentration 1 mg/mL in 0.2 M acetate buffer at pH 5.5 containing 0.17 M NaCl) was added to 1 mL of the extract. The sample was left for 15 min at room temperature and then centrifuged (5,000 × *g*). After 15 min of centrifugation, the supernatant was removed and the pellet was washed with 1 mL of acetate buffer and centrifuged again (5,000 × *g*/15 min). The pellet was dissolved in 4 mL of solution containing: 1% sodium dodecyl sulphate (SDS) and 5% triethanolamine (TEA), and 1 mL of 0.01 M FeCl_3_ in 0.01 M HCl was added. The samples were left at room temperature for 30 min and the absorbance was measured at 510 nm. The results were expressed as absorbance units at 510 nm per 1 mg of the extract (A_510_/mg).

### 3.7. UV Spectra

The UV spectra of phenolic compounds present in the extracts were recorded using a Beckman diode UV-Vis Beckman DU 7500 diode array spectrophotometer.

### 3.8. Total Antioxidant Capacity (TAC)

The total antioxidant capacity of the seeds was determined according to the Trolox equivalent antioxidant activity (TEAC) assay described by Re *et al*. [32] with some modifications. Briefly, ABTS radical cation was prepared by passing a 5 mM ABTS aqueous solution through the oxidising reagent, manganese dioxide, on a Fisher Brand P8 filter paper. Excess manganese dioxide was removed from the filtrate by passing the solution through a 0.2 mm Fisher Brand membrane. The extracts were diluted in 5 mM phosphate buffered saline (PBS, pH 7.4), to an absorbance of about 0.700 (±0.020) at 734 nm in a 1-cm cell. 1.0 mL of each of the extracts was added to 5 mL ABTS^•+^ solution and the absorbance reading were taken 10 min after the initial mixing at room temperature. PBS was used as the blank. The calibration curve of Trolox standard was plotted. The antioxidant capacities of the seeds were expressed as mmol Trolox equivalent content in 1 g FW. TAC was expressed as mmol Trolox equivalent/g of extract.

### 3.9. Antiradical Activity of Seed Extracts against the DPPH Radical

The scavenging effect phenolics from the extracts was monitored as described by Yen and Chen [26]. A 0.1 mL methanolic solution containing between 0.1–0.5 mg of an was mixed with 2 mL of methanol. Then added to a methanolic solution of DPPH^●^ (1 mM, 0.25 mL). The mixture was vortexed for 1 min, left to stand in the dark at room temperature for 20 min, and absorbance of the solution was then measured at 517 nm with the spectrophotometer. As the control sample solution of BHA (synthetic antioxidant) was used.

### 3.10. Reducing Power of the Extracts

The reducing power of phenolics present in the extracts was determined as described by Oyaizu [33]. A suspension of extract (0.1–0.5 mg) in 1 mL of deionized water was mixed with 2.5 mL of 0.2 M phosphate buffer (pH 6.6) and 2.5 mL of 1% (w/v) potassium ferrocyanide. The mixture was incubated in a water bath at 50 °C for 20 min. Following this, 2.5 mL of 10% trichloroacetic acid solution was added and the mixture was then centrifuged at 1,750 × *g* for 10 min. A 2.5-mL aliquot of the upper layer was mixed with 2.5 mL of deionized water and 2.5 mL of 0.1% (w/v) FeCl_3_ in distilled water was added to 2.5 mL of this sample. After 10 minutes, the absorbance of the mixture was measured at 700 nm. As a control sample solution of ascorbic acid was used.

### 3.11. Separation and Analysis of Phenolic Acids by HPLC

Phenolic acids (free and those liberated from soluble esters and from soluble glycosides) were isolated from the extracts according to the method previously described by Weidner *et al*. [34]. An aqueous suspension of the methanol extract (200 mg in 20 mL) was adjusted to pH 2 with 6 M HCl, and free phenolic acids were extracted five times into 20 mL of diethyl ether using a separatory funnel. The ether extract was evaporated to dryness under vacuum at room temperature. The water solution was neutralized and then lyophilized. The residue was dissolved in 20 mL of 2 M NaOH and hydrolyzed for 4 h under a nitrogen atmosphere at room temperature. After acidification to pH 2 using 6 M HCl, phenolic acids released from soluble esters were extracted from the hydrolyzate five times into 30 mL diethyl ether. Nine milliliters of 6 M HCl were added to the water solution and the solution was placed under a nitrogen atmosphere and hydrolyzed for 1 h in a boiling water bath. Phenolic acids released from the soluble glycosides were separated from the hydrolyzate five times into 45 mL of diethyl ether. After ether evaporation, the dry residue was dissolved in 2 mL methanol and filtered through a 0.45 µm nylon filter. The sample was injected onto an HPLC column. A Shimadzu HPLC system was employed and comprised a LC-10 ADVP pump, photodiode array detector UV-VIS SPD-M10AVP, oven CTO-10 ASVP, Controller SCL-10AVP. The conditions of the separations were as follows: prepacked LUNA C_18_ column (5 µm, 4.6 × 250 mm; Phenomenex); mobile phase water-acetonitrile-acetic acid (88:10:2, v/v/v) [35]; flow rate of 1 mL/min; injection volume of 20 µL; the detector was set at 280 and 320 nm; oven temperature was 20 °C.

### 3.12. Analysis of Catechins with HPLC

The same HPLC system was used for catechin analysis. The separation was performed in a gradient system: solvent A: water-acetonitrile-acetic acid (93:5:2, v/v/v); solvent B: water-acetonitrile-acetic acid (58:40:2, v/v/v); linear gradient from 0 to 100% B for 50 min [36]; flow rate of 1 mL/min; injection volume of 20 µL; the detector was set at 280 nm; oven temperature was 20 °C.

### 3.13. Statistical Analysis

All experiments were repeated four times, with three replications for each sample. The results reported as the means ± SD. Statistically significant differences in the mean values were tested by Student’s *t*-test.

## 4. Conclusions

The analysed seeds of grapevines contained large quantities of total phenolics and tannins, as well as observable amounts of catechin, epicatechin, gallic, *p*-coumaric, caffeic, and ferulic acids. The total content of phenolic compounds and tannins in the seeds of *V. vinifera* was about two-fold higher than in *V. coignetia*. The extracts from seeds of *V. vinifera* exhibited greater antioxidant activity than those of *V. coignetiae*. The total contents of phenolic compounds and tannins in acetone extracts were higher than those in methanolic ones. Acetone extracts also exhibited stronger antiradical properties, as well as stronger reducing power. The acetone extract of *V. vinivera*, due to high antioxidant activity, can be applied for production of nutraceuticals and for employment in functional foods. According to literature, data on this extract should also offer strong biological activity.
